# Investigating the spatiotemporal patterns and clustering of attendances for mental health services to inform policy and resource allocation in Thailand

**DOI:** 10.1186/s13033-024-00639-5

**Published:** 2024-05-09

**Authors:** Chawarat Rotejanaprasert, Papin Thanutchapat, Chiraphat Phoncharoenwirot, Ornrakorn Mekchaiporn, Peerut Chienwichai, Richard J Maude

**Affiliations:** 1 Department of Tropical Hygiene, Faculty of Tropical Medicine, Mahidol University, Bangkok, Thailand; 2 Mahidol-Oxford Tropical Medicine Research Unit, Faculty of Tropical Medicine, Mahidol University, Bangkok, Thailand; 3 Princess Srisavangavadhana College of Medicine, Chulabhorn Royal Academy, Bangkok, Thailand; 4 Department of Computer Engineering, Faculty of Engineering, King Mongkut’s University of Technology Thonburi, Bangkok, Thailand; 5 Centre for Tropical Medicine and Global Health, Nuffield Department of Medicine, University of Oxford, Oxford, UK; 6 The Open University, Milton Keynes, UK

**Keywords:** Mental health, Equality, Health system, Spatial, Temporal, Thailand

## Abstract

**Background:**

Mental illness poses a substantial global public health challenge, including in Thailand, where exploration of access to mental health services is limited. The spatial and temporal dimensions of mental illness in the country are not extensively studied, despite the recognized association between poor mental health and socioeconomic inequalities. Gaining insights into these dimensions is crucial for effective public health interventions and resource allocation.

**Methods:**

This retrospective study analyzed mental health service utilization data in Thailand from 2015 to 2023. Temporal trends in annual numbers of individuals visiting mental health services by diagnosis were examined, while spatial pattern analysis employed Moran’s I statistics to assess autocorrelation, identify small-area clustering, and hotspots. The implications of our findings for mental health resource allocation and policy were discussed.

**Results:**

Between 2015 and 2023, mental health facilities documented a total of 13,793,884 visits. The study found anxiety, schizophrenia, and depression emerged as the top three illnesses for mental health visits, with an increase in patient attendance following the onset of the COVID-19 outbreak. Spatial analysis identified areas of significance for various disorders across different regions of Thailand. Positive correlations between certain disorder pairs were found in specific regions, suggesting shared risk factors or comorbidities.

**Conclusions:**

This study highlights spatial and temporal variations in individuals visiting services for different mental disorders in Thailand, shedding light on service gaps and socioeconomic issues. Addressing these disparities requires increased attention to mental health, the development of appropriate interventions, and overcoming barriers to accessibility. The findings provide a baseline for policymakers and stakeholders to allocate resources and implement culturally responsive interventions to improve mental health outcomes.

**Supplementary Information:**

The online version contains supplementary material available at 10.1186/s13033-024-00639-5.

## Background

Mental illnesses have a significant impact on global public health, with a high number of years lived with disability (YLDs) and disability-adjusted life years (DALYs) [1, 2]. In Thailand, mental health problems in adolescents are increasing, and the country is struggling to evaluate the mental health situation accurately [3]. Approximately 20% of the Thai population is affected by mental illness [4], yet access to effective prevention and therapy services remains limited due to stigma and prejudice [5]. The COVID-19 pandemic has worsened social inequality in Thailand, with health inequality becoming a major public health concern [6].

While the association between poor mental health and socio-economic inequalities is widely acknowledged [7], the geographical distribution of mental illness in Thailand has been insufficiently explored. This oversight can result in disproportionately low budgets, consequently leading to less effective mental health services. The frequently inadequate health budgets for mental illness, relative to the disease burden, stem from prevailing stigma and prejudice especially in low- and middle- income countries (LMICs) [8, 9]. This financial disparity is exacerbated by the association of stigma with resources, which are disproportionately allocated for mental disorders. Furthermore, the relationship between equity and mental health highlights the necessity for a comprehensive understanding of mental health distribution and associated factors.

Research indicates that mental disorders exhibit a spatial structure influenced by factors with an uneven geographical distribution [10, 11]. The knowledge of geographical distribution can yield significant benefits, including enhancing access to and the quality of existing mental health care. Additionally, it can contribute to a prevention agenda aimed at reducing the population burden of mental disorders through targeted action on social, structural, and political determinants of mental health. Therefore, gaining a more thorough understanding of various aspects of the epidemiology of mental illness, including space-time dimensions, is crucial. This knowledge is indispensable for informing public health interventions and guiding investments to improve health services at both national and sub-national levels [7].

There has been growing research interest in the impact of residential location on mental health [7, 12]. Identifying areas with a higher risk of disease may provide important clues to better understand the mechanisms contributing to social disparities [12]. Although the majority of people with mental disorders reside in low- and middle-income countries, including Thailand [13], there is a relative dearth of mental health research conducted in these settings, particularly concerning space-time dimensions. Investigating the spatiotemporal distribution of mental health services and their accessibility is essential to raise awareness of the burden of mental illnesses and guide resource allocation for the development of effective mental health services.

In this study, our objective was to analyze the spatiotemporal distribution of mental health facility visits recorded in Thailand from 2015 to 2023. Employing space-time analyses incorporating cluster detection techniques and correlation matrices we retrospectively examined the annual number of patients attending mental health services across provinces, as documented by the Department of Mental Health, Ministry of Public Health (MOPH). The investigation of the spatiotemporal distribution of mental health services and their accessibility is vital for gaining insights into the mental illness situation and guiding resource allocation to enhance mental health services. Our study’s findings may contribute valuable information for policymakers and aid in the targeted development of mental health services across the country.

## Methods

### Mental health system, study design and data sources

#### Mental health services in Thailand

Mental health services in Thailand have undergone significant transformation since their inception in 1889, marked by the establishment of the first psychiatric hospital, followed by the subsequent development of numerous regional hospitals [14]. A pivotal moment occurred in 1977–1978 with the “Monitoring Mental Health Needs” project in collaboration with the World Health Organization (WHO), which underscored the necessity for community mental health services [15]. Consequently, mental health services expanded beyond psychiatric hospitals to integrate with the public healthcare system, a transition solidified by the integration of mental health into primary care in 1982 [16]. Presently, Thailand boasts 13 public health regions, each equipped with accessible health services, including the capital city of Bangkok.

At the core of Thailand’s mental health infrastructure are 13 regional mental health centers operating under the Department of Mental Health, Ministry of Public Health. These centers play a crucial role in facilitating community mental health provision by liaising with local healthcare networks [14]. Additionally, mental/psychiatric hospitals, some medical school hospitals, and military hospitals provide inpatient psychiatric care, alongside outpatient services [16]. While the majority of psychiatric beds are situated within mental/psychiatric hospitals, with 6.26 beds per 100,000 population and 126.27 annual admissions per 100,000 population [16], government hospitals are increasingly expanding their mental health services to meet the growing demand [15]. Furthermore, Thai mental health service provision includes outpatient facilities situated within mental hospitals, medical school hospitals, and military hospitals, along with 720 community/nonhospital mental health outpatient facilities and 378 other outpatient facilities, such as mental health daycare or treatment centers, ensuring accessible care for diverse mental health needs nationwide [15].

#### Study design and data sources

A retrospective analysis of reported numbers of individuals visiting mental health services in in each year by diagnosis was conducted using data collected from the health data center (HDC), Department of Mental Health, Ministry of Public Health (MOPH). Notably, health facility visits from the mental health surveillance system were employed due to the challenges associated with estimating mental disorder prevalence in Thailand. These data were aggregated by year and notified during the years 2015 to 2023, aggregated at the provincial level, based on location of residence. The list of ICD-10 disorders included in this study is provided in supplementary document S1.

Population statistics for Thailand were obtained from the National Statistical Office (NSO). The geographic coordinates and provincial boundary data were obtained from the GEO package file in the Global Administrative Region Database (GADM), a high-resolution territorial database containing provincial down to subdistrict level data for the whole world. Data files for analysis were compiled using the Python programming language version 3.6. To determine the annual attendance rate for each mental illness, the number of reported attendances was divided by the mid-year population for each province. The resulting data were analyzed using descriptive statistics to determine the attendance rates of mental illness in each province.

### Spatial pattern analysis

Spatial analyses in epidemiology often require the investigation of spatial variation in mapped variables to appropriately selected analytical tools [17]. To assess the presence and strength of spatial autocorrelation over the whole study area and test the spatial independence assumption in the spatial pattern analysis, we used the global Moran’s I statistic. Furthermore, to examine the local behavior and identify locations of hot-spots and outliers, we used the local Moran’s I statistic to detect small-area clustering of mental illness.

#### Spatial contiguity matrices

In order to calculate spatial autocorrelation, a measure of contiguity is required to specify the neighborhood structure. The weight matrix, $\varvec{W}$, is an adjacency matrix that calculates the distance between province pairs $\left(i,j\right)$by conditions as spatial weights ${w}_{ij}$ are 1 when $i$ and $j$ are neighbors, otherwise 0. In this study, the weight matrix was created using the queen criterion, which is appropriate for provinces that are connected by sharing a common edge or a common vertex [18, 19]. The queen criterion is the most suitable method for the Thai provinces, considering their interconnectivity. By using the weight matrix, we can calculate the spatial autocorrelation measures, which help to identify spatial clustering of mental illness in the study area.

#### Global spatial correlation

To assess the presence and strength of spatial autocorrelation over the entire study area, we used the global Moran’s I statistic. This statistic measures the spatial autocorrelation of a variable by considering both the location and magnitude features. The formula for calculating the global Moran’s I is as follows

$$I=\frac{n}{{\sum }_{i=1}^{n}{\sum }_{j=1}^{n}{w}_{ij}}\frac{{\sum }_{i=1}^{n}{\sum }_{j=1}^{n}{w}_{ij}\left({x}_{i}-\overline{x}\right)\left({x}_{j}-\overline{x}\right)}{{\sum }_{i=1}^{n}{\left({x}_{i}-\overline{x}\right)}^{2}} i\ne j$$

where *n* is the number of provinces, ${x}_{i}$ and ${x}_{j}$ are the variable of interest at locations of $i$ and $j$, $\stackrel{-}{x}$ is the arithmetic mean and ${w}_{ij}$is the spatial weight value in the spatial matrix between locations of $i$ and $j$ [20]. The global Moran’s I ranges from − 1 to 1. A value close to 1 indicates that neighboring positions with similar values tend to cluster, while a value close to -1 indicates that neighboring positions with dissimilar values tend to cluster. A value close to 0 suggests that the data are randomly distributed. When the p-value is statistically significant, it indicates that the null hypothesis should be rejected, and adjacent positions tend to cluster similarly. However, the global Moran’s I can only measure overall spatial autocorrelation and cannot identify local behaviors. Further investigation is required using the local Moran’s I to explore spatial patterns at a finer scale.

#### Local spatial detection

The local Moran statistic is useful for evaluating the spatial autocorrelation of health outcomes at the local level. It can help identify spatial clusters and outliers within a region of interest [18]. The local Moran can be written at spatial unit $i$ as

$${I}_{i}=\frac{n\left({x}_{i}-\overline{x}\right){\sum }_{j=1}^{n}{w}_{ij}\left({x}_{j}-\overline{x}\right)}{{\sum }_{i=1}^{n}{\left({x}_{i}-\overline{x}\right)}^{2}}$$

The results can be categorized into four types: hotspot (HH), coldspot (LL), high-low (HL), and low-high (LH) outliers. Hotspots and coldspots (HH and LL) indicate disease clusters, while LH and HL outliers indicate low-prevalence and high-prevalence locations that are surrounded by the opposite [20]. By examining a Moran scatter plot, the locations with significant clustering can be easily identified. This method is particularly useful for identifying local spatial patterns, which cannot be captured by the global Moran’s I statistic alone.

#### Spearman’s rank correlation coefficient

Spearman’s Rank Correlation Coefficient was employed to assess the correlation between mental illnesses at each location, offering insights into the relationships among these diseases. This information proves valuable for understanding patterns and facilitating resource allocation at a more granular level. The Spearman’s correlation coefficient is a nonparametric measure of a non-linear and monotonic relationship between two ranked variables. In this study, the variables mean the disorder, so we used the Spearman’s correlation coefficient to measure a relationship between pairs of disorders. This method returns values between − 1 and 1. For interpretation, if the value is closer to + 1 or -1, the two disorders will have a more monotonic relationship to each other [21, 22]. A value of -1 means a perfect negative association between two disorders. A value of 1 means a perfect positive association of disorders. A value of 0 means there is no association between two disorders. The coefficient is used when variables are ordinal or variables aren’t normally distributed [23]. The statistical equation is written as

$${R}_{s}=1-\frac{\left(6{\sum }_{i=1}^{n}{d}_{i}^{2}\right)}{n\left({n}^{2}-1\right)}$$

where *R*_*s*_ is spearman’s rank correlation coefficient, ∑*d*^2^_i_ is a sum of the squares between x-variable and y-variable ranks, and n is the number of data points of two disorders [24].

## Results

### Descriptive analysis and mapping

**Fig. 1:**
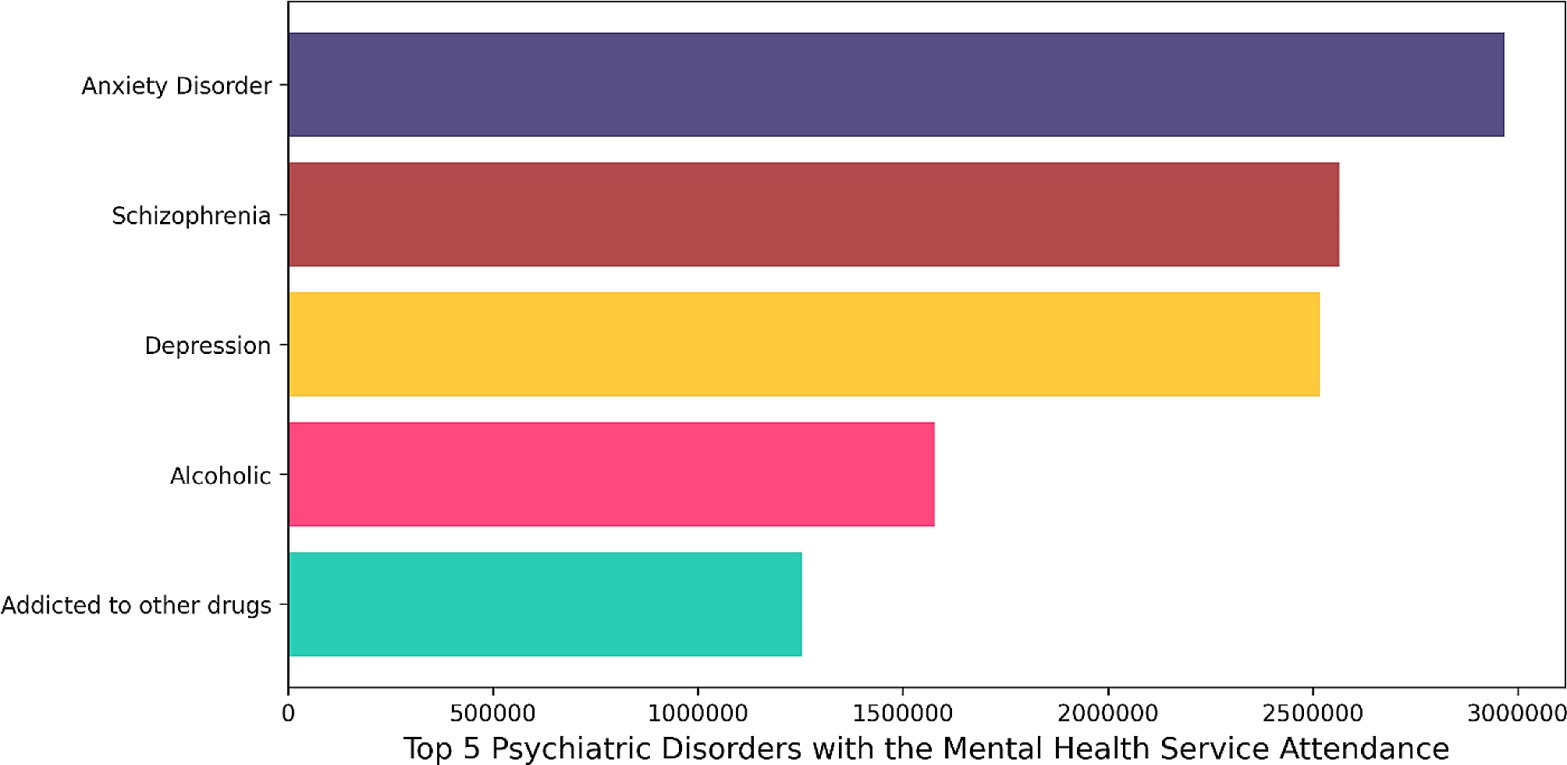
The five most common mental health diagnoses and total visits at governmental mental health services in Thailand from 2015 to 2023

**Fig. 2:**
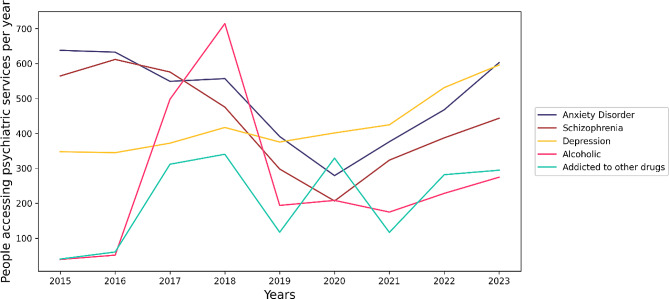
Trends in annual patient visits per 100,000 population at governmental mental health services by diagnosis in Thailand from 2015 to 2023

From 2015 to 2023, a total of 13,793,884 cases visited mental health facilities as recorded in the Department of Mental Health’s data system. Figure 1 highlights the top five mental health illnesses with the highest number of patients accessing services during the study period. Notably, anxiety, schizophrenia, and depression emerged as the most prevalent disorders, with 2,966,629, 2,565,052, and 2,518,348 individuals attending mental health services, respectively. Figure 2 provides insights into the annual trends of the top 5 mental health diagnoses, emphasizing the changing patterns over the years. The observed increase in patient attendance after the COVID-19 pandemic underscores the growing demand for mental health services, a phenomenon that may be influenced by various factors, including the impact of the COVID-19 pandemic on mental well-being.

**Fig. 3:**
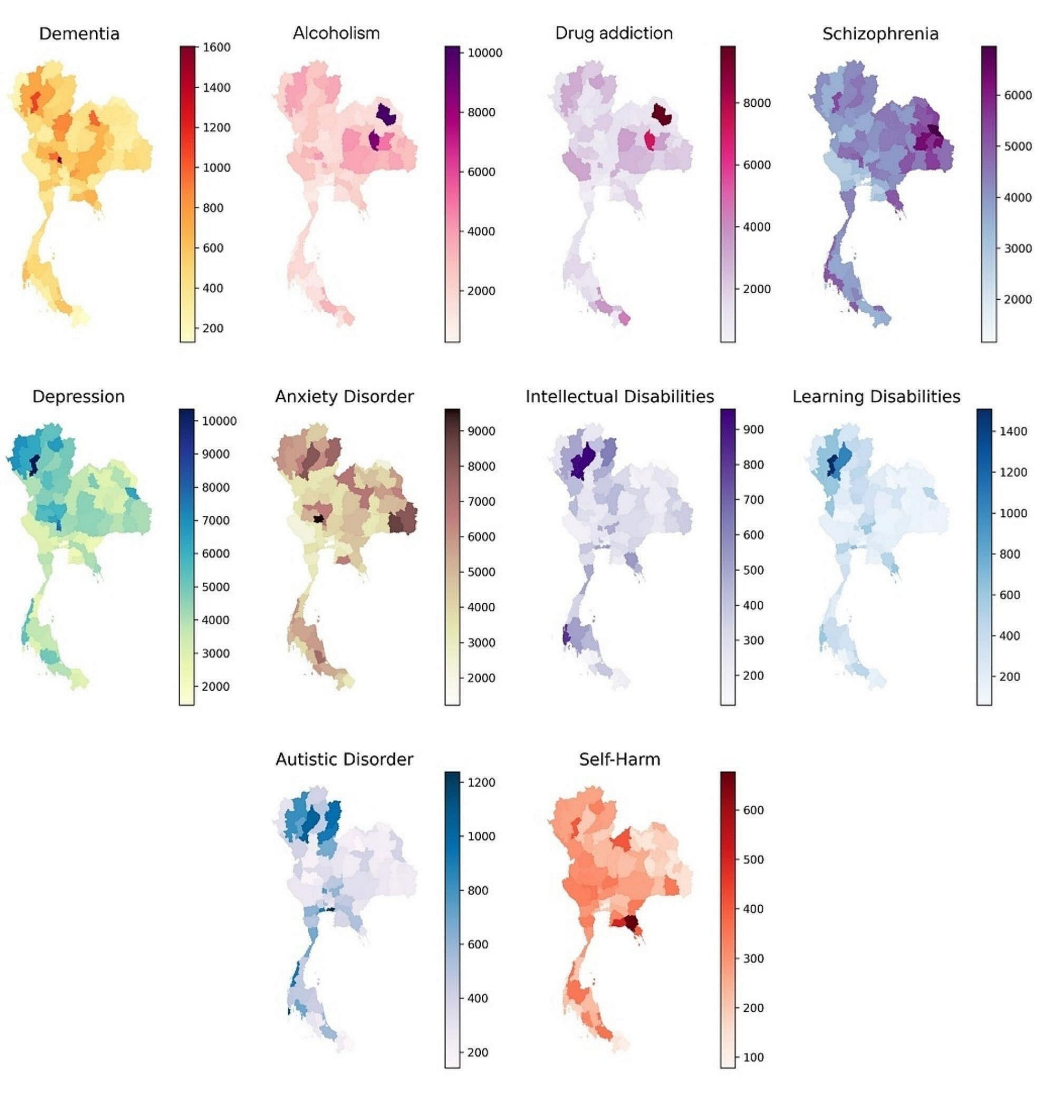
Total numbers of individuals visiting mental health services by diagnosis and province per 100,000 population from 2015 to 2023

Figure 3 shows province-level maps of attendance rates per 100,000 population for various mental health conditions diagnosed between 2015 and 2023. The shading indicates the rate for each disorder, with darker areas representing higher attendance rates. The northeastern region of Thailand exhibited higher numbers attending for alcoholism, drug addiction, anxiety, and schizophrenia than other regions. The north had the highest numbers attending for depression, anxiety, intellectual and learning disabilities, while self-harm was more common in the north and provinces along international borders.

### Spatial pattern analysis

#### Global and local spatial detection

To assess the overall spatial autocorrelation, we employed the global Moran’s I test. The statistical estimate and p-value of the global Moran’s I hypothesis test for each mental condition.

are shown in Table 1. Except for alcoholism and drug addiction, all mental disorders had significant global test statistics with a p-value below 0.05.

**Table 1:** The estimates and p-values of global Moran’s I hypothesis test of total individuals visiting mental health services by disorder in Thailand during 2015–2023

Disorder	I statistics	*P*-value	Interpretation
Dementia	0.228913	0.002	clustered
Alcoholism	0.136254	0.036	clustered
Drug addiction	-0.018491	0.492	scattered
Schizophrenia	0.544651	0.001	clustered
Depression	0.298251	0.001	clustered
Anxiety Disorder	0.295847	0.001	clustered
Intellectual Disabilities	0.223837	0.003	clustered
Learning Disabilities	0.324229	0.001	clustered
Autistic Disorder	0.207196	0.006	clustered
Self-Harm	0.295195	0.001	clustered

**Fig. 4:**
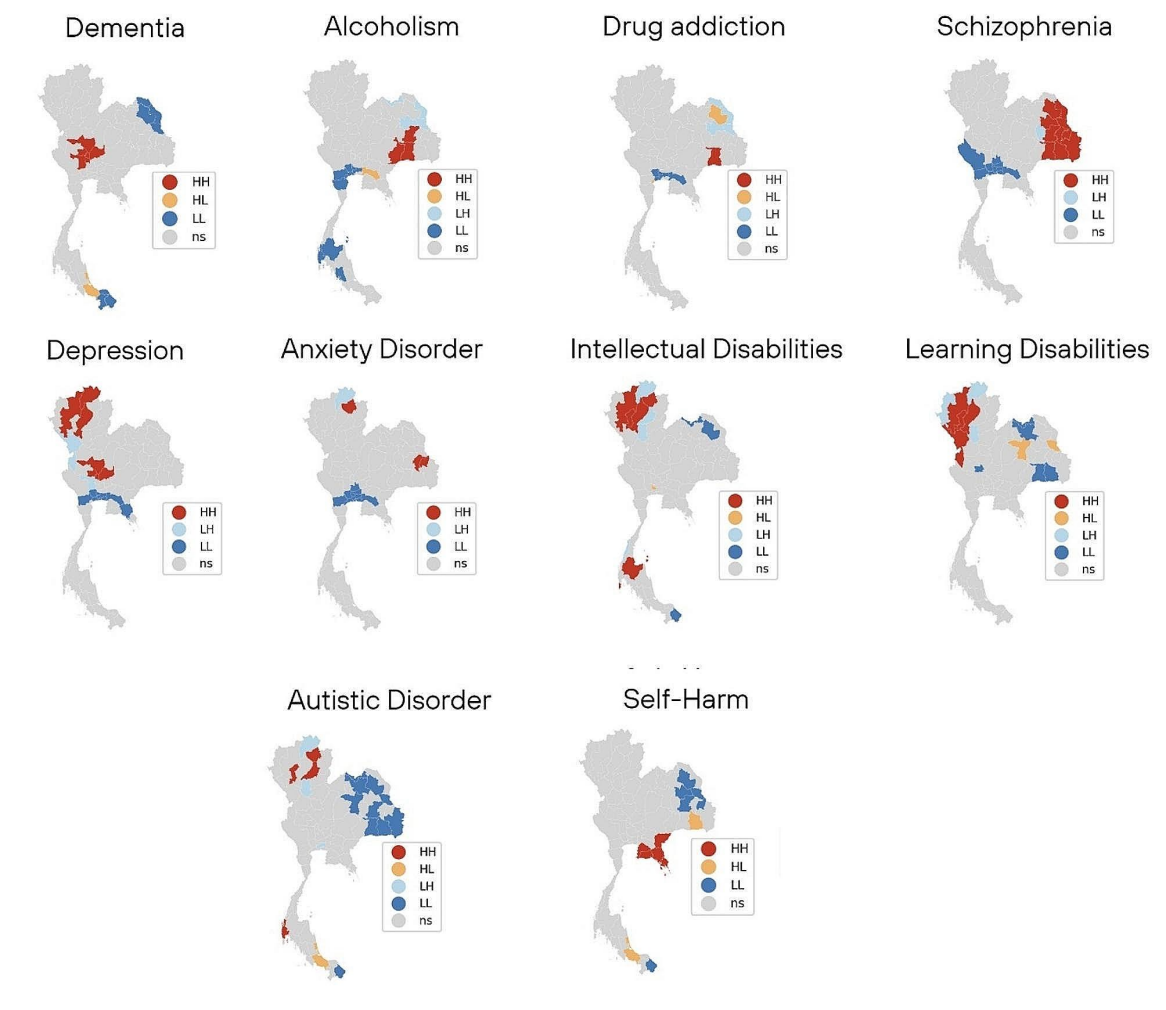
Province level maps of sum of individuals visiting mental health services during the study period for mental illness clusters in Thailand using the local Moran’s I statistic

To further investigate local patterns of disease clustering and outliers, we employed the local Moran’s I (LISA) cluster technique for each mental disorder. The resulting maps of disease clusters and outliers are presented in Fig. 4. High-High (HH) provinces were denoted by red, High-Low (HL) by yellow, Low-High (LH) by sky blue, Low-Low (LL) by blue, and not significant areas were labeled by gray. Our analysis revealed that the northern region had areas of high contact levels for five disorders, including depression, anxiety, intellectual disabilities, learning disabilities, and autistic disorder. In the northeast region, there were clusters of four disorders: alcoholism, drug addiction, anxiety, and schizophrenia. The central region mainly exhibited a cluster of depression. The southern region had two clusters: intellectual and learning disabilities. The eastern region exhibited a cluster of alcoholism and self-harm, while we also found clusters for intellectual and learning disabilities in the west. Additional details regarding the spatial clusters identified for mental health disorders can be found in Table S2 in the supplementary document, which offers an overview of the spatial clusters detected for various mental health disorders based on the local Moran results.

### Spatial mental disorder correlation

**Fig. 5:**
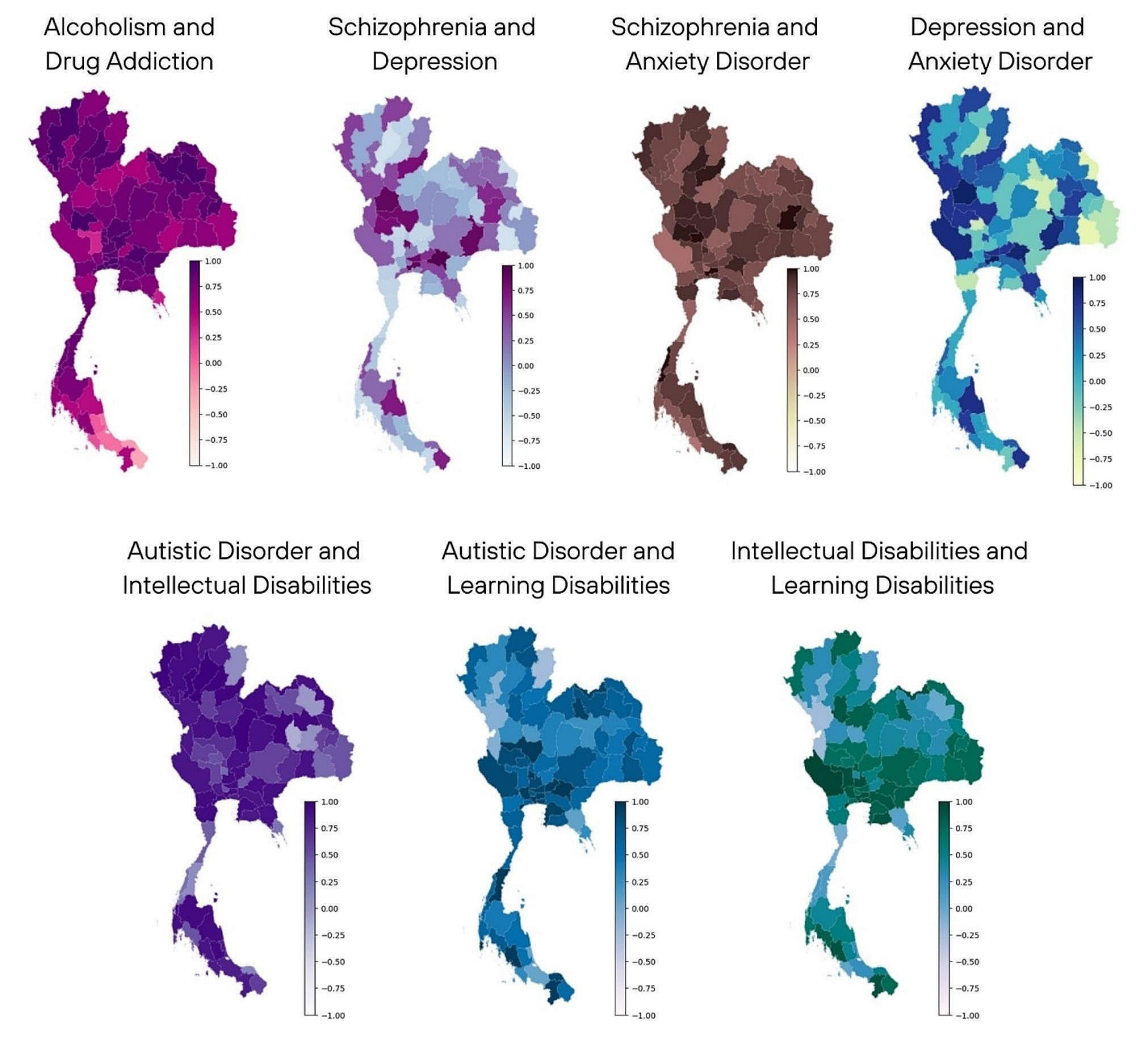
Provincial-level maps showing Spearman’s correlation coefficients for pairs of mental disorders during the study

We conducted an analysis to examine the correlations between pairs of mental disorders at a provincial level using Spearman’s correlation coefficient. Figure 5 illustrates the maps of Spearman’s correlation for various disorder pairs across Thailand. Our findings revealed several positive correlations between disorder pairs in specific regions. In the northern region, alcoholism and drug addiction, as well as schizophrenia and anxiety, exhibited positive correlations across most provinces. Additionally, there was a notable correlation between autistic disorder and intellectual and learning disabilities in this region. Similarly, in the northeast region, alcoholism and drug addiction, along with schizophrenia and anxiety, showed positive correlations in most provinces.

Positive relationships were also observed in the central and western regions, particularly between depression and anxiety. In the western and eastern regions, there was a positive correlation between schizophrenia and depression, as well as depression and anxiety disorders. These findings suggest the existence of shared risk factors or comorbidities among these disorder pairs in specific regions of Thailand. It’s important to note that while these correlations provide valuable insights, they do not directly indicate the importance or level of risk associated with each disorder pair. Rather, they reflect the similarity in patterns of disorder pairing across different locations. Differences in the magnitude of paired disorders can result in variations in correlation coefficients, as observed in specific provinces across Thailand.

## Discussion

In low- and middle-income countries, such as Thailand, notable disparities persist in the treatment and attendance rates for mental illnesses [25]. While health system factors, particularly financial and medical resources, critically influence the coverage and effectiveness of mental health service interventions, mental health systems in these countries remain insufficiently investigated. The global consequences of the COVID-19 pandemic have cast a shadow on public mental health. In Thailand, the observed decline in the number of patients seeking mental health services during the pandemic is potentially associated with the adaptation of psychiatric facilities and outpatient departments for outbreak management. This adaptation may have deterred individuals with mental health concerns from accessing health facilities due to heightened infection-related anxieties [26]. Nevertheless, amid the ongoing COVID-19 pandemic, the intersection of equality and poverty has emerged as a crucial factor influencing the increasing prevalence of mental health problems, particularly in low- and middle-income countries, underscoring the escalating concerns related to mental health and psychosocial well-being in Thailand and these regions [27–29].

Moreover, studies have shown the pandemic’s worsening impact on psychopathological and socioeconomic factors, contributing to a new burden of mental illnesses [30–32]. These circumstances highlight the imperative to reorganize existing mental health services, addressing unmet needs and preparing for post-pandemic challenges, especially in LMICs [33]. Addressing high levels of unmet need is acknowledged to be extremely complex, as outlined by the World Mental Health Report, which urgently calls for the transformation of mental health and underscores collaborative efforts among various stakeholders, including professionals [34].

Despite the central role of promoting mental health and preventing mental health conditions in the public mental health approach, this domain remains under-researched and challenging to change [35]. The report navigates the uncertainty surrounding the approach to social determinants of mental health, delineating roles and responsibilities between the health sector and other domains [34]. Additionally, the transformation of mental health services necessitates the integration of mental health considerations into discussions addressing social determinants such as poverty alleviation and violence prevention [35].

The findings of this study underscore the spatiotemporal heterogeneity in the attendance rates for mental illness in Thailand, reflecting a complex interplay of factors. Assessment of unmet need has been conducted across diverse and vulnerable populations, including minority ethnic groups and individuals grappling with specific health conditions, notably mental illnesses [36]. In 2019, the Thai Ministry of Public Health reported that there were only 25 community-based psychiatric units with 0.4 beds per 100,000 population, limiting the accessibility of minority groups such as hill tribe people [36]. Our study also identified several areas of significance for mental health in the north of Thailand, where most hill tribes are located in rural areas, particularly along the borders of neighboring countries.

The hill tribe communities in Thailand may face specific challenges related to mental health, which could include various factors such as cultural practices, certain health behaviors, economic conditions, and educational resources [25, 37]. Additionally, a significant portion of hill tribe individuals may experience limited access to healthcare services and medical assistance [38]. Moreover, the existing mental health screening tools available in peripheral healthcare units in remote areas might not be fully tailored to the specific needs of the hill tribe communities. Therefore, it is plausible that the prevalence of mental health issues in these communities could be underrepresented in the available data. Consequently, there is a need for an enhanced healthcare system to more accurately identify mental health concerns and provide appropriate care and monitoring, with a particular focus on addressing depression. Public health interventions should be directed at improving awareness about mental health among hill tribe communities, exploring culturally suitable methods or channels for communication in local hill tribe languages [38].

Through community-based participatory research, a depression care model tailored for the hill tribe population was developed and demonstrated clear effectiveness when tested in a hill tribe community [39]. This model can be applied to other hill tribe communities in northern Thailand to enhance depression care. Additionally, the stateless and hill tribe population in Thailand relies on accessing all public services, including healthcare [40]. Stigma significantly impacts their lives, given their existence as an invisible population and the negative treatment they receive. Addressing this issue through access to public health resources and education is considered effective under the implementation schemes of relevant organizations. Furthermore, stigma related to drug use has multifaceted impacts on physical and mental health, influenced by personal traits, community dynamics, and socio-economic factors, including culture and tribes. Implementing a program to reduce drug and substance use among hill tribes holds the potential to minimize this stigma [41].

Another region with disease clusters was the northeast, which is the largest region of the country consisting of 20 provinces, where many migrant workers from neighboring countries live. Most of these migrants reside in semi-urban and semi-rural communities, where they might encounter various environmental problems that put them under stress. The socioeconomic factors prevalent in this region may also contribute to the development of depressive symptoms in many individuals without access to proper assistance [42]. Additionally, there has been an increase in illegal drug use in Thailand [43], causing extensive economic and social problems, with the northeastern part seen as a drug trafficking area [43, 44].

A nationally representative face-to-face household survey was conducted in the USA using a fully structured diagnostic interview. The results revealed that anxiety disorders were the most prevalent, affecting almost 20% of the population, followed by mood disorders, impulse control disorders, and substance use disorders. These findings suggest a similar pattern of mental illness prevalence in Thailand. In addition, the study identified a strong correlation between anxious depression (major depressive episode with generalized anxiety disorder) and comorbid substance disorders (both alcohol abuse and dependence with drug abuse and dependence) [45].

Moreover, common mental disorders, such as depression and anxiety, significantly contribute to the burden of disease and disability in low- and middle-income countries [46]. Investigating the social determinants of mental disorders in LMICs, including Thailand, is crucial as recognized key factors such as poverty, low education, social exclusion, gender disadvantage, conflict, and disasters are major determinants of mental disorders in LMICs [47]. Thus, research exploring the impact of these determinants on mental health outcomes in Thailand can inform targeted interventions and policies, mitigating risk factors associated with mental disorders. This effort can also contribute to a comprehensive understanding of prevalence and associated risk factors, guiding the development of effective strategies to enhance mental well-being of the population in the country.

Nevertheless, the imperative task of reducing the costs associated with mental illness is a highly complex issue, compounded by the challenge of addressing substantial unmet needs. Considering the limitations of current treatment options for certain diagnoses, it becomes important to explore potential protective factors unique to specific groups. Within the Thai cultural context, the communal living patterns in rural villages foster robust social networks, potentially enabling individuals with mental disabilities to lead successful lives. Studies suggested that, with the support of family and community, along with medication, self-regulation, essential self-management skills, and occasional intervention by psychiatric-mental health practitioners, those living with schizophrenia can engage in daily activities, work, and contribute to the well-being of others [48, 49]. Thus, leveraging the support of families and communities can serve an alternative social resource in the rehabilitative process of mental health support in Thailand.

There are several limitations to this study, including the potential stigmatization of mental disorders, which might prevent patients from seeking treatment altogether. The stigmatization of mental illness can be influenced by factors such as media and education [50]. Moreover, the number and location of mental health services in each province may affect how easily people can access the service, leading to an underestimation of the actual number of patients with mental illness. The analysis used the data available from the Thai government which did not include the numbers of new diagnoses by condition. Instead, the available metric was annual numbers of individuals visiting mental health services. Thus, conditions with longer duration of more frequent episodes for which the same person attends services over multiple years e.g., schizophrenia may be over-represented compared to conditions with shorter duration or less frequent episodes.

In our study, we aimed to explore the spatiotemporal distribution of visits at mental health facilities. However, it is important to acknowledge a limitation in our correlation analysis, where the pairing of disorders may seem unconventional. While the correlations observed in our results are based on real-world data, it is important to recognize that the reported number of visits may be influenced by various factors. Despite these unconventional pairings, our findings offer exploratory insights into the utilization patterns of mental health services, which can inform hypotheses for future research. Additionally, we acknowledge that the Spearman spatial maps may not directly inform complex resource allocation decisions. While our research provides valuable insights into mental health service utilization patterns, future studies could enhance our findings by incorporating additional factors such as demographic characteristics, socioeconomic status, and healthcare infrastructure to develop more nuanced models for resource allocation.

Moreover, there are overarching challenges in estimating the prevalence of mental disorders and mental health-related service contacts. These estimates are crucial for policy formulation, research, advocacy, and resource allocation. However, the burden of mental illness may be potentially underestimated at various geographical locations and scales [2, 51]. Contributing to this underestimation are socio-economic factors, including stigma and prejudice, particularly pronounced in LMICs [8, 52]. An alternative approach involves combining various data sources to enhance the accuracy of estimates, although there is currently no standardized method for this [53]. Nonetheless, it is important to note that the main analysis focused on the relative spatial distribution rather than comparing absolute measures of disease occurrence between diagnoses, potentially limiting the impact of this aspect on the findings. Despite these acknowledged limitations, this study provides valuable evidence that can serve as a baseline for addressing the high need and persistent scarcity of financial resources, workforce, and infrastructure for mental health services in the country.

## Conclusions

This study highlighted the significant spatiotemporal heterogeneity of mental disorders in Thailand and identified service gaps for vulnerable populations and socioeconomic issues in the country. The findings underscore the need for increased attention to mental health in Thailand and the development of appropriate interventions to address these disparities. Overcoming the barriers to improving accessibility to mental health services will require social enlightenment, political will, and a public movement. These findings provide a valuable information for policymakers and stakeholders to allocate resources and develop effective interventions that are responsive to the local cultural context and diverse needs of the population. It is our hope that this study will contribute to improving the quality of mental health services and treatments in Thailand, ultimately leading to improved mental health outcomes for all.

### Electronic supplementary material

Below is the link to the electronic supplementary material.

Supplementary Material 1

## Data Availability

The data that support the findings of this study were publicly available. The reported number of individuals visiting mental health mental health services was collected from the website of health data center, Department of Mental Health, Ministry of Public Health while population statistics for Thailand were obtained from the website of National Statistical Office.
