# Supplementary material for: Investigating the spatiotemporal patterns and clustering of attendances for mental health services to inform policy and resource allocation in Thailand
Source: Int J Ment Health Syst. 2024 May 9;18:19. doi: 10.1186/s13033-024-00639-5 (PMC11080131; doi:10.1186/s13033-024-00639-5)
Supplement: Supplementary file 1 — Supplementary Material 1 [file 13033_2024_639_MOESM1_ESM.docx]

**Supplementary document: Investigating the spatiotemporal patterns and clustering of attendances for mental services to inform policy and resource allocation in Thailand**

**Supplementary Table S1: Description of ICD-10 mental disorder code**

| **Disorders** | **ICD-10 Code** |
| --- | --- |
| Dementia | F00-F03 |
| Alcoholism | F10 |
| Drug addiction  (excluding amphetamine addiction) | F11, F12, F13, F14, F16, F17, F18, F19 |
| Schizophrenia | F20 |
| Depression | F32, F33, F341, F38, F39 |
| Anxiety Disorder | F40-F48 |
| Intellectual Disabilities | F70-F79 |
| Learning Disabilities | F81 |
| Autistic Disorder | F84 |
| Self-Harm | X60-X84 |

**Supplementary Table S2:** Spatial clusters of different disorders based on the local Moran index, categorized into High-High (HH), High-Low (HL), Low-High (LH), and Low-Low (LL) classifications.

| **Disorders** | **High-High (HH)** | **High-Low (HL)** | **Low-High (LH)** | **Low-Low (LL)** |
| --- | --- | --- | --- | --- |
| Alcoholism | Northeast | East | Northeast | West and South |
|  |  |  |  |  |
| Anxiety Disorder | Northeast and North | - | North | Western and Central |
|  |  |  |  |  |
| Autistic Disorder | North and South | South | North | Northeast and South |
|  |  |  |  |  |
| Dementia | Central | South | - | South and Northeast |
|  |  |  |  |  |
| Depression | Central and North | - | West | West, Central and East |
|  |  |  |  |  |
| Drug Addiction | Northeast | Northeast | Northeast | Central and East |
|  |  |  |  |  |
| Intellectual Disabilities | North and South | Central | North | South and Northeast |
|  |  |  |  |  |
| Learning Disabilities | North and West | Northeast | North | Northeast and Central |
|  |  |  |  |  |
| Schizophrenia | Northeast | - | Northeast | West and Central |
|  |  |  |  |  |
| Self-Harm | East | Northeast and South | - | Northeast and South |
